# A temporary measure for ureteral stenting

**DOI:** 10.1308/rcsann.2025.0047

**Published:** 2025-05-20

**Authors:** R Doodnath, B Rampersad

**Affiliations:** Department of Paediatric Surgery, Eric Williams Medical Sciences Complex Compound, Champs Fleurs, Trinidad and Tobago

## Background

Ureteral stents are used routinely in elective and emergency urological surgery to reduce stricture formation. A challenge may arise in the paediatric population when even the smallest stent cannot be passed through the vesico-ureteric junction (VUJ) to allow adequate drainage of the kidney. One must then consider other options for anastomotic stenting.

## Technique

**Figure 1:**
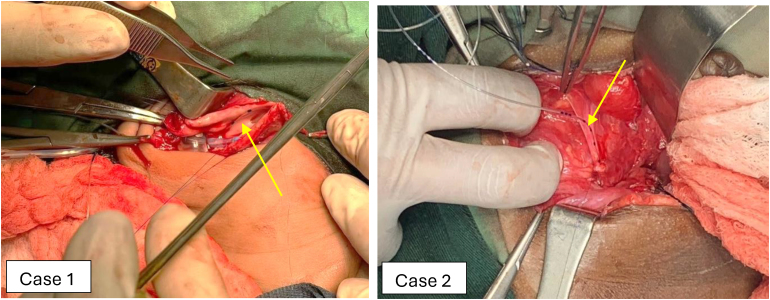
Epidural catheter being inserted distally as shown by the yellow arrow

Two paediatric cases required intraoperative ureteral stenting. The smallest stent available, 3Fr, would not advance through the VUJ. Case 1 was a 3-day-old term infant with massive right-sided hydronephrosis secondary to a pelvi-ureteric junction obstruction. This patient also had a right nephrostomy. Case 2 was an 18-month-old baby undergoing a pull-through procedure for Hirschsprungs disease with an iatrogenic right ureteric injury. The ureters were repaired with 6/0 PDS sutures and a 19G epidural catheter was used to function as a trans-anastomotic stent (Figure 1). This was easily advanced through the VUJ. In case 1, the proximal end was attached to the nephrostomy tube and in case 2 the distal end was attached to the urinary catheter to prevent migration. This was left in situ for 6 weeks and 10 days, respectively. Both children had very good outcomes.

## Discussion

Use of an epidural catheter as a ureteric stent worked well because these tubes are soft, with adequate markings for easy identification of length in the renal pelvis and bladder, and are easy to remove. An epidural catheter serves as a good option for temporary drainage of the kidney while allowing ureteric healing, without the need for further surgical procedures.

Stenting, especially in very a small anastomosis, is effective in reducing stricture formation. The alternative is to try retrograde insertion via cystoscopy; however, this is unlikely to be successful because the VUJ is usually tight in these cases. Long-term drainage such as nephrostomy or ureterostomy can lead to infection and make further reconstruction challenging. Hence we recommend temporary stenting with an epidural catheter if traditional stents cannot be used.

